# MiR-15b can target insulin receptor to regulate hepatic insulin signaling in mice

**DOI:** 10.1080/19768354.2019.1583125

**Published:** 2019-02-27

**Authors:** Wei-Dong Li, Jin-Rong Xia, Yan-Shu Lian

**Affiliations:** a Department of Gastroenterology, Zhongda Hospital, School of Medicine, Southeast University, Nanjing, Jiangsu Province, People’s Republic of China; b Department of Preventive Medicine, Jiangsu health vacation College, Nanjing, Jiangsu Province, People’s Republic of China

**Keywords:** miR-15b, insulin receptor, insulin resistance, type II diabetes

## Abstract

Now diabetes is growing to be a health problems globally. However, its specific pathogenesis still needs further exploration. Here we showed that miR-15b was upregulated in the palmitate-induced HepG2 cells and livers of hyperglycemic mice. At the same time, we confirmed that the insulin receptor was a direct target of miR-15b. Then we found that the manipulation of miR-15b expression level could affect the insulin signaling pathway of HepG2 cells and the inhibition of miR-15b in liver of ob/ob mice can improve insulin sensitivity of mice. Furthermore, our study demonstrated that palmitate could upregulate the expression of miR-15b by activating PPARα. Our findings established PPARα-responsive miR-15b as a critical regulator of hepatic insulin signaling, thus serving as a new potential therapeutic target for diabetes.

## Introduction

Diabetes is a rapidly growing global health problem that is closely related to the pathogenesis of metabolic syndrome (Boren et al. 2013). Type 2 diabetes is characterized by hyperglycemia and decreased response of peripheral tissues to insulin (Kahn et al. 2006; Petersen and Shulman 2006). Hepatic insulin resistance is one of the most important causes for the development of type 2 diabetes. In the state of insulin resistance, the liver's response to insulin is reduced, while gluconeogenesis is increased and glycogen synthesis is reduced simultaneously, eventually leading to hyperglycemia in the body (Samuel and Shulman 2012). Therefore, studies on the complex network of liver insulin signaling pathway can help us to understand the molecular mechanism of hepatic insulin resistance and type 2 diabetes and provide new solutions for the treatment of metabolic diseases.

IR (insulin receptor), one of the receptor tyrosine kinase family, is an important node in the insulin signaling pathway. Insulin activates downstream signaling pathway by binding to IR on the cell membrane and autophosphorylation of IR. Mice with systemic knockout IR exhibit hyperinsulinemia, hyperglucoemia and ketoacidosis, and die soon after birth (Joshi et al. 1996). Consistently, liver-specific knockout IR mice also show hyperglycemia, hyperlipidemia, hyperinsulinemia and obesity (Michael et al. 2000). Furthermore, Won-Mo Yang *et al* have reported that mir-195 which was induced by saturated fatty acid could impair insulin signaling and glycogen metabolism in HepG2 cells though targeting IR (Yang et al. 2014). These reports indicate that direct or indirect regulation of IR expression can effectively regulate the insulin signaling pathway.

MicroRNA is a non-coding RNA consisting of 19∼25 nucleotides that regulates the expression of a target gene by binding to its 3′ Untranslated Region (UTR) of the target gene (Bartel 2009). Many studies have demonstrated that mircroRNA played an important role in regulating insulin signaling pathway (Pandey et al. 2011; Trajkovski et al. 2011; Rottiers and Näär 2012; Liu et al. 2014). Although more and more microRNAs have been identified to be able to regulate hepatic glucose metabolism, different mircroRNA play different roles in the regulation of hepatic insulin signaling pathway by targeting different genes. Therefore, it is necessary to discover new mircroRNA that regulate insulin signaling pathway in order to comprehensively understand the mechanism of diabetes. Many studies have shown that mircroRNA-15b (miR-15b) was involved in the regulation of many important biological activities of the body. For example, studies have shown that miR-15b regulated cell apoptosis in CD5+ B cells of blood of human chronic B lymphoblastic leukemia patients (Cimmino et al. 2005; Yue and Tigyi 2010) and gastric cancer cell line SGC7901/VCR (Xia et al. 2008) by targeting bcl-2. Moreover, a study by Sun et al. showed that miR-15b regulated insulin synthesis by targeting UCP-2 in MIN6 cells (Sun et al. 2011). Similarly, Tingming Liang et al. found miR-15b was significantly elevated in the liver of ob/ob mice through deep sequencing (Liang et al. 2013). However, whether miR-15b is directly involved in maintaining the homeostasis of hepatic glucose still needs further exploration.

Our study here demonstrated miR-15b which was induced by palmitate regulated the insulin signaling pathway in HepG2 cells by targeting insulin receptor in HepG2 cells. We also found that inhibition of miR-15b in ob/ob mice could improve insulin sensitivity in ob/ob mice. We also demonstrated palmitate could induce the expression of miR-15b by activating PPARα. These findings led us to propose that palmitate-responsive hepatic miR-15b is a critical regulator of glucose homeostasis, which could be a new potential therapeutic target for type II diabetes.

### Methods & Materials

#### In vivo study

All mice were males aged 8–12 weeks and were maintained at a temperature of 23 ± 3°C and a humidity of 35 ± 5% under a 12 h dark/light cycle in a specific pathogen-free animal facility. High fat diet (HFD) mouse was fed with 60% high fat diet (Research Diets D12492) for 16 weeks. All experimental animals were free to drink water. The tissues were immediately excised into liquid nitrogen and stored in −80°C. The blood was also collected for the purpose of detecting the components in the serum.

#### Tolerance tests

In the insulin resistance test, mice were starved for 6 h to inject insulin (0.8 U/ kg) into the mice, and the blood glucose of the mice was measured at 0, 15, 30, 60, 90 and 120 min, respectively.

#### Insulin signaling analysis

HepG2 cells were transfected with mimics, plasmids for 36 h, starved for 12 h, and then treated with 100 nM insulin for 5–20 min to collect protein.

#### Plasmids and RNA oligonucleotide

To construct reporter plasmids, the 3′ UTR sequence of IR containing MRE (primer-F: 5′-AGGGTTGGGCTTTGAGAAGGTTT-3′ primer-R: 5′-CACCACTGCTCCCAAAGAAATAC-3′)was cloned into the p-RL-TK plasmid. Mutations in miR-15b regulatory elements (MRE) was made by using KOD-Plus mutagenesis kit (Toyobo) following to the manufacturer’s instruction. The 2 kb fragment upstream of miR-15b was amplified and cloned into PGL3Basic plasmid (primer-F: 5′-TCTGCCAGGGTGCAAGGC-3′; primer-R: 5′- TTTGAGGCAGCACAGTATGGC-3′). The CDS region of insulin receptor (primer-F:5′-ATGGGCTTCGGGAGAGGATGT-3′; primer-R:5′- TTAGGAAGGGTTTGACCTTG-3′) and PPARα (primer-F: 5′-ATGGTGGACACAGAGAGCC-3′; primer-R: 5′-TCAGTACATGTCTCTGTAGA-3′) were cloned into the pcDNA3.1 plasmid. The shRNA (5′- TGCTGTTGACAGTGAGCGACCAGTGCATGTCCGTGGAGACTAGTGAAGCCACAGATGTAGTCTCCACGGACATGCACTGGCTGCCTACTGCCTCGGA-3′) of PPARα was cloned into MSCV-LMP plasmid. Mimics and AntagomiR is a specially-labeled and chemically modified double-stranded small RNA that modulates the biological function of target genes by mimicking endogenous miRNAs. Mimics for miR-15b (5′-UAGCAGCACAUCAUGGUUUACA-3′) and AntagomiR for miR-15b (5′-UGUAAACCAUGAUGUGCUGCUA-3′) were obtained from Genepharma. Nonsense sequences were used as mimics control (5′-UCACAACCU CCUAGAAAGAGUAGA-3′) and Ant control (5′-UCUACUCUU UCUAGGAGG UUGUGA-3′). According to the in vivo experiment, briefly, We divided the ob/ob mice into two groups and injected AntagomiR-NC and AntagomiR-15b (5ug/g) through the tail vein every two days. After three injections, we performed ITT test to the mice.

#### Palmitate treatment

For palmitate-induced insulin resistance, the cells were treated with BSA-conjugated palmitate (0.5 mM) for 30 h. In the Luciferase assay palmitate treated HepG2 cell with 0.5 or 1 mM.

#### Luciferase assay

HepG2 cells were planted in a 48-well plate and co-transfected with pRL-TK and pGL3basic for internal reference and every group was treated differently. 48 h after transfection, cells were lysed and relative luciferase activity was analyzed with the Dual Luciferase Reporter Assay System (Promega) on a luminometer (Promega).

#### Real Time-PCR

RNA was isolated using Trizol reagent from cells, 500 ng of RNA was reverse-transcribed into cDNA using the RT system by PrimeScript RT reagent Kit (TaKaRa RR037A). Real Time PCR which contain 4.6 ul cDNA,5ul SYBR GREEN (Roche 4887352001) and 0.4ul (10uM) primers was performed on ABI 7500 Fast Real Time PCR system.

#### Western blotting

Cells or tissues were lysed in RIPA lysis buffer containing 10 mM Tris-HCl (pH7.5), 1% SDS, 1 mM Na3VO4, 10 mM NaF and protease inhibitor cocktail (Roche 4693132001). Protein samples (10 ug) were used for immunoblotting and separated on 80 V constant pressure SDS-PAGE. Then the protein were transferred onto nitrocellulose membranes with 300 mA for 3 h and blocked by 5% skim milk then probed with the various antibodies at 4°C overnight. On the second day, the membranes were washed with TBST buffer and probed with secondary antibodies which was diluted with 5% skim milk at 1:1500. Detection was performed by measuring the chemiluminescent signal as assayed by SuperSignal Ultra. Antibodies for AKT, p-AKT (Ser473), IR, p-IR (Cell Signaling Technology,1:1000), GAPDH (Sigma-Aldrich 1:5000) diluted with 5% BSA were used for western blotting.

#### Statistical analysis

Data were expressed as means ± SEM. The statistical differences in mean values were assessed by Student’s t test. All experiments were performed at least twice, and representative data are shown.

## Results

### miR-15b was upregulated in the palmitate-induced HepG2 cells and livers of hyperglycemic mice

**Figure 1.**
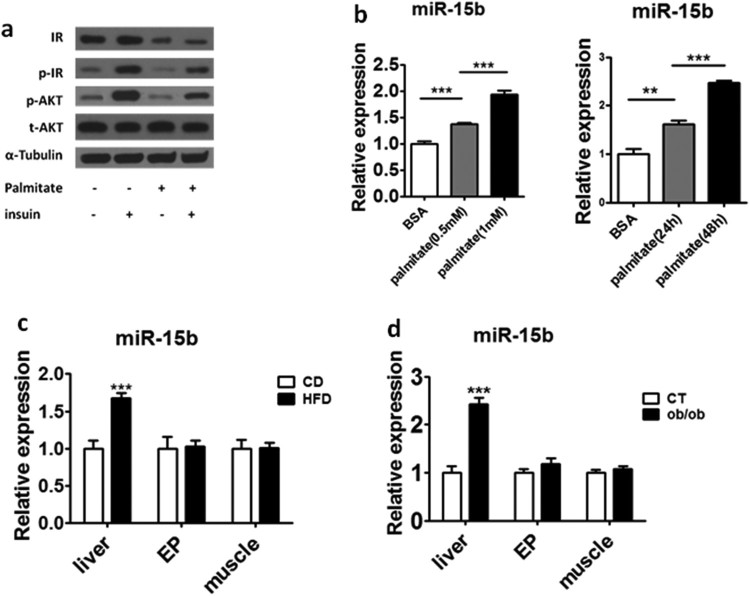
miR-15b was upregulated in the palmitate-induced HepG2 cells and hyperglycemic mice livers. (a) The protein level of (IR and AKT) and phosphorylation (p-IR and p-AKT) of insulin signaling intermediates were analyzed by immunoblot. (b) The expression level of miR-15b was examined with the increasing doses of palmitate (left) and treatment duration (right). HepG2 cells were incubated with or without palmitate (0.5, 1 mM) for 48 h. HepG2 cells were incubated with 0.5 mM palmitate for 24 h or 48 h. (c) The expression of miR-15b in liver, EP and muscle were quantified by qRT-PCR from mice fed with CD (chow diet) and HFD (high fat diet). (d) The expression of miR-15b in liver, EP and muscle were quantified by qRT-PCR from ob/ob and wild type mice. Means ± s.e.m. are shown.*P < 0.05, **P < 0.01 and ***P < 0.001 (Student’s t-test). All experiments were repeated at least twice and representative results are shown.

To explore the mechanism of hepatic insulin resistance, we used palmitate (PA) to treat HepG2 cells to establish a model of cellular insulin resistance. As shown in Figure 1(a), we found the protein level of p-IR and p-AKT decreased significantly after palmitate treatment. At the same time, we also found palmitate caused a decrease in IR protein level in HepG2 cells. According to previous reports, miR-15b which was significantly elevated in the liver of ob/ob mice (Liang et al. 2013) was able to target IR according to bioinformatics predictions. Therefore we tested the expression level of miR-15b, and we found PA could increase the expression level of miR-15b in a dose-dependent and time-dependent manner (Figure 1(b)). We further examined the expression level of miR-15b in liver, epididymal visceral adipose (EP) and muscle in HFD and ob/ob mice, and we found miR-15b expression was significantly increased in the liver of HFD and ob/ob mice, but not in EP and muscle (Figure 1(c–d)). These results suggested miR-15b may play an important role in regulating insulin signaling in the liver of mice.

### MiR-15b can directly target insulin receptor in HepG2 cells

**Figure 2.**
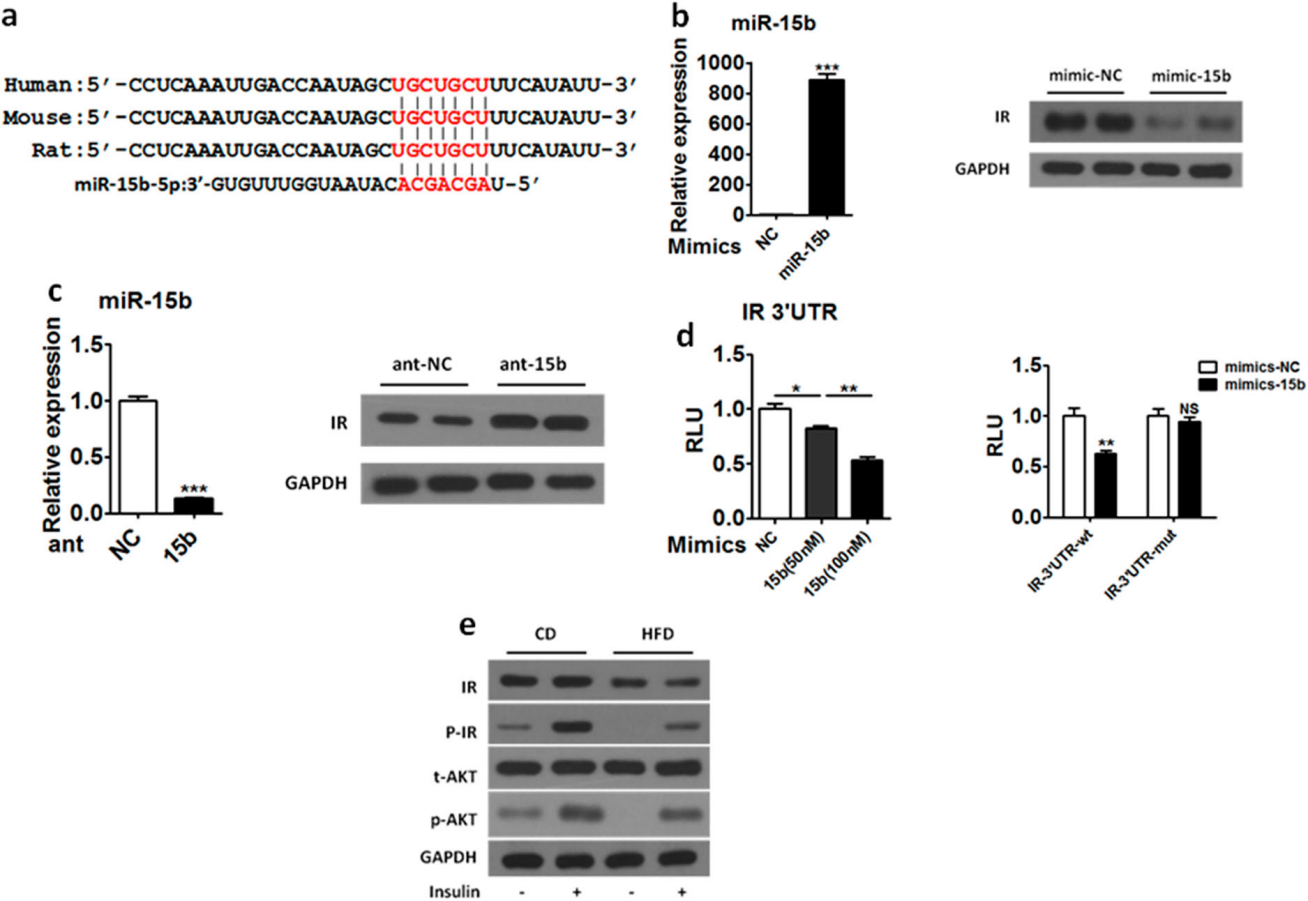
MiR-15b can directly target IR. (a) Sequence alignment of the 3′-UTR of the IR in different organisms and miR-15b reveals a miR-15b response element (MRE). Seed sequences are highlighted in red. (b) The expression of miR-15b in the HepG2 cells transfected with mimics-NC (100 nM) or mimics-15b (100 nM) (left). Western blot analysis of IR in the HepG2 cells transfected with mimics-NC (100 nM) or mimics-15b (100 nM) (right). (c) The expression of miR-15b in the HepG2 cells transfected with Ant-NC (100 nM) or Ant-15b (100 nM) (left). Western blot analysis of IR in the HepG2 cells transfected with Ant-NC (100 nM) or Ant-15b (100 nM) (right). (d) The activity of the reporter containing the 3′-UTR of IR was determined in HepG2 cells in the presence of increasing amounts of miR-15b mimics (left). Reporters with mutated IR-3′ UTRs were also analyzed (right). (e) Mice fed CD and HFD were fasted overnight and sacrificed 20 min after i.p. injections of vehicle or insulin (0.8U/kg body weight). The protein level of IR, p-IR, t-AKT and p-AKT of insulin signaling intermediates in the liver were analyzed by immunoblotting. Means ± s.e.m. are shown.*P < 0.05, **P < 0.01 and ***P < 0.001 (Student’s t-test). All experiments were repeated at least twice and representative results are shown.

We found there was a miR-15b binding site on the 3′ UTR of IR through bioinformatics analysis and the binding site was conserved among species (Figure 2(a)). To further explore whether IR is the target gene of miR-15b, we transfected mimics-15b and mimics-NC in HepG2 cells and we found mimics-15b successfully overexpressed miR-15b (Figure 2(b) left) and IR's protein level decreased significantly (Figure 2(b) right). Consistent with result shown in Figure 2(b), we transfected Ant-NC and Ant-15b in HepG2 cells, and we found Ant-15b can significantly suppress the expression of miR-15b (Figure 2(c) left), meanwhile the IR’s protein level were significantly increased (Figure 2(c) right). In order to confirm whether IR is a direct target for miR-15b, we cloned the 3′ UTR, including MRE, of IR into PRL-TK plasmid, and luciferase reporter assays demonstrated that mimics-15b reduced the activity of the reporter gene containing IR 3′ UTR-WT in a dose-dependent manner (Figure 2(d) left) without altering the activity of the reporter gene containing IR 3′ UTR-mut (Figure 2(d) right). We also examined IR protein level in the liver of HFD and chow diet (CD) groups, and we found IR protein level and insulin-stimulated phosphorylation of IR were significantly decreased in the liver of the HFD group than in the CD group (Figure 2(e)). These observations clearly indicated that IR was a direct target gene of miR-15b in the liver.

### Regulation of miR-15b expression level could modulate the insulin signaling pathway of HepG2 and insulin sensitivity in ob/ob mice

**Figure 3.**
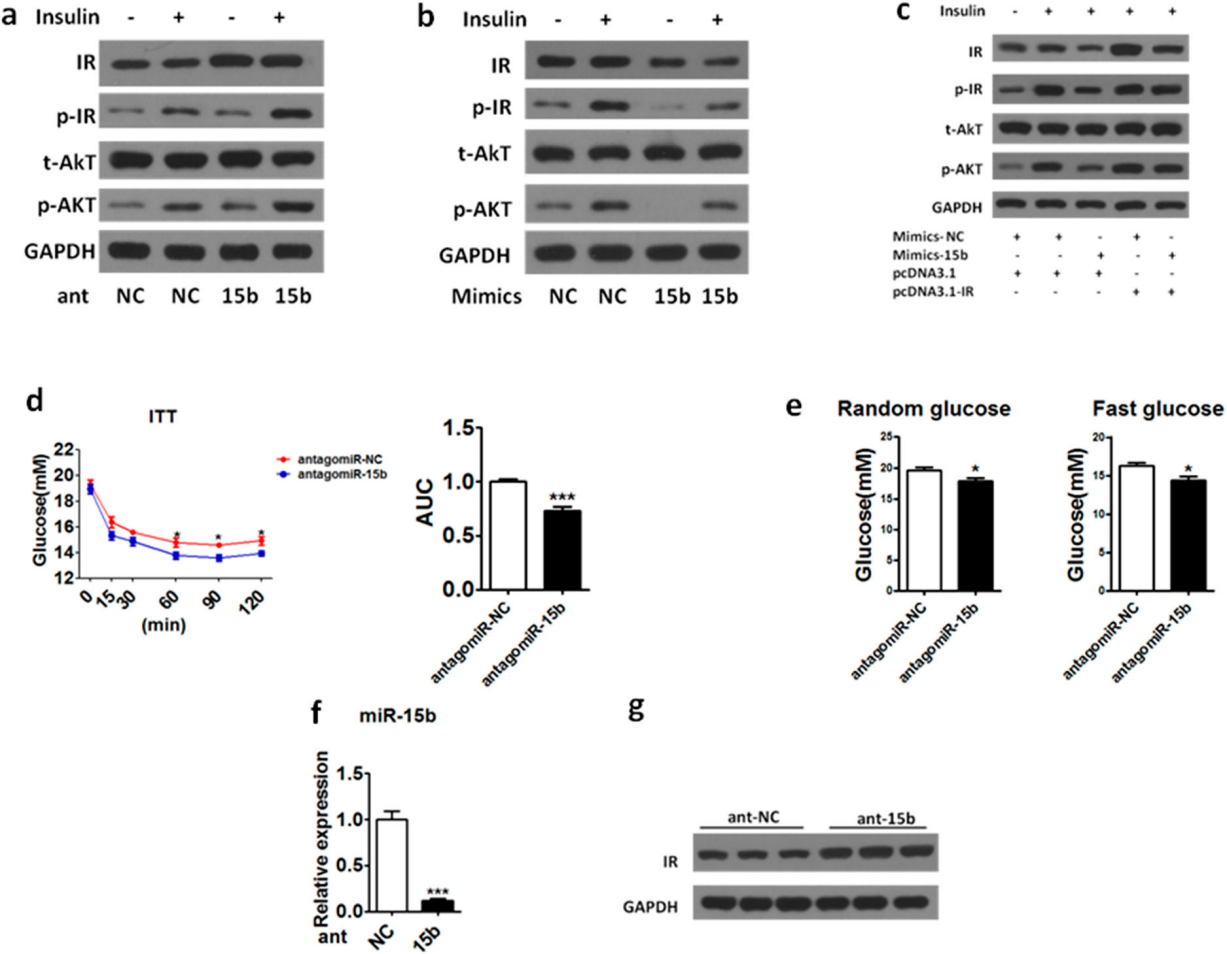
Regulation of miR-15b expression level can affect insulin signaling in HepG2 cells and glucose homeostasis in ob/ob mice. (a) Western blot analysis IR, p-IR, p-AKT and AKT in the insulin-stimulated HepG2 cells transfected with mimics-NC (100 nM) or mimics-15b (100 nM). (b) Western blot analysis IR, p-IR, p-AKT and AKT in the insulin-stimulated HepG2 cells transfected with Ant-NC (100 nM) or Ant-15b (100 nM). (c) Western blot analysis of IR, p-IR, p-AKT and AKT in HepG2 cells transfected with mimics-15b or pcDNA3.1-IR, or both mimics-15b and pcDNA3.1-IR after insulin administration. (d) Insulin-tolerance test (ITT) were performed in the ob/ob mouse tail vein injection with AntagomiR-NC or AntagomiR-15b (left). Area under the curve (AUC) data ITT were calculated (right). (e) The random glucose (left) and fast glucose (right) was evaluated in the ob/ob mice treated with AntagomiR-15b or AntagomiR-NC respectively. (f) The expression level of miR-15b in the liver of AntagomiR-NC-injected and AntagomiR-15b-injected ob/ob mice. (g) The protein level of IR in the livers of AntagomiR-NC-injected and AntagomiR-15b-injected ob/ob mice. Means ± s.e.m. are shown.*P < 0.05, **P < 0.01 and ***P < 0.001 (Student’s t-test). All experiments were repeated at least twice and representative results are shown.

In order to confirm whether the expression of miR-15b affect hepatic insulin signaling, we investigated the insulin signaling in HepG2 cells after miR-15b level was overexpressed by mimics-15b. As shown in Figure 3(a), we found that overexpression of miR-15b decreased IR protein level and the insulin-stimulated phosphorylation of IR and AKT. Consistently, knockdown of miR-15b by Ant-15b enhanced the IR protein level as well as the phosphorylation level of IR and AKT (Figure 3(b)). Similarly, the suppressive effect of mimics-15b on the IR and phosphorylation of IR and AKT were totally abrogated after the restoration of IR expression in HepG2 cells (Figure 3(c)). Those results showed that manipulating the expression of miR-15b can indeed affect the insulin signaling pathway in HepG2 cells. We would like to further explore whether regulation of miR-15b expression in liver can affect insulin sensitivity in mice. We divided the ob/ob mice into two groups and injected AntagomiR-NC and AntagomiR-15b through the tail vein every two days. We performed ITT on mice after three times of injection and we found that the insulin sensitivity of the Ant-15b group was significantly stronger than that of the Ant-NC group (Figure 3(d)). Consistently, we found the random and fast glucose of Ant-15b group was both lower than that of the Ant-NC group (Figure 3(e)). Further, we examined the expression level of miR-15b and the protein level of IR in mouse liver. We found that the expression level of miR-15b was significantly decreased (Figure 3(f)) and the protein level of IR was significantly increased (Figure 3(g)) in the liver of Ant-15b group compared to Ant-NC group. Those results indicated that suppression of miR-15b expression level could enhance the insulin signaling pathway of HepG2 and insulin sensitivity in ob/ob mice.

### Palmitate may induce the expression of mir-15b by activating PPARα

**Figure 4.**
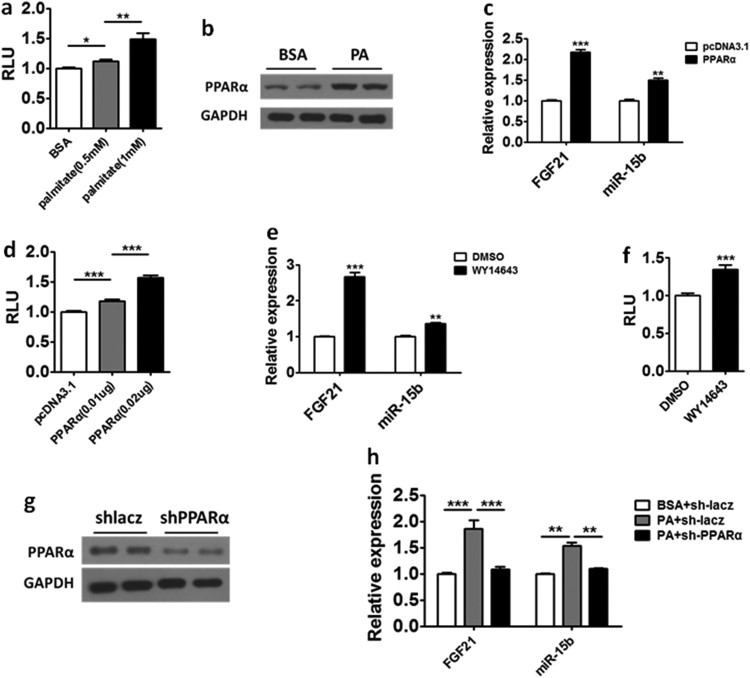
Palmitate may upregulate the expression of miR-15b by activating PPARα. (a) The activity of the reporter containing the promoter of miR-15b was determined in HepG2 cells treating with BSA and increasing palmitate dose. (b) The protein level of PPARαin HepG2 cells treated with BSA or palmitate. (c) The mRNA level of FGF21 and miR-15b was evaluated in HepG2 cells transfected with pcDNA3.1 or PPARα. (d) The activity of the reporter containing the promoter of miR-15b was determined in HepG2 cells transfected with pcDNA3.1 or increasing PPARα dose. (e) The mRNA level of FGF21 and miR-15b was evaluated in HepG2 cells transfected with DMSO or WY14643 (100 nM). (f) The activity of the reporter containing the promoter of miR-15b was determined in HepG2 cells treated with DMSO or WY14643 (100 nM). (g) The protein level of PPARα was compared in HepG2 cells transfected with sh-lacz or shPPARα. (h) The mRNA level of FGF21 and miR-15b was compared in HepG2 cells treated with BSA, palmitate or both palmitate and sh-PPARα. Means ± s.e.m. are shown.*P < 0.05, **P < 0.01 and ***P < 0.001 (Student’s t-test). All experiments were repeated at least twice and representative results are shown.

We want to further explore the molecular mechanism by which PA regulates miR-15b expression. Therefore, we cloned a 2 kb DNA fragment upstream of miR-15b into the PGL3Basic plasmid to study PA’s effect on miR-15b’s promoter activity. As shown in Figure 4(a), the PA can increase the activity of the reporter gene containing miR-15b promoter in a dose-dependent manner. Based on the previous results, we hypothesized PA may up-regulate the expression level of miR-15b by promoting the binding of a transcription factor to the promoter of miR-15b. Many studies have reported PA is one of the major ligands for PPARα (Forman et al. 1997; Kliewer et al. 1997). Therefore, to further investigate whether PPARα can promote miR-15b expression,we cloned the mouse PPARαinto pcDNA3.1 plasmid, which can produce a high level of PPARαprotein (Figure 4(b)). Then pcDNA3.1-PPARα or control pcDNA3.1 plasmides were transfected into HepG2 cells respectively and we found PPARα overexpression could significantly upregulated miR-15b and FGF21 which was regulated by PPARα (Badman et al. 2007) (Figure 4(c)). We also found PPARα can increase the activity of the reporter gene containing miR-15b promoter in a dose-dependent manner (Figure 4(d)). Consistently, WY14643, an agonist of PPARα, was able to upregulate the expression of miR-15b (Figure 4(e)) and increase the activity of the reporter gene (Figure 4(f)). We also used shRNA to knock down the expression level of PPARα (Figure 4(g)) and then we found shRNA down-regulated the expression level of FGF21 and miR-15b (Figure 4(h)). These results showed that PA may up-regulate miR-15b by activating PPARα.

## Discussion

Insulin resistance leads to higher susceptibility to various metabolic diseases, especially type II diabetes. The liver is one of the main peripheral organs that maintain the homeostasis of glucose metabolism in the body. Therefore, exploring the mechanisms of the hepatic insulin resistance could help us to provide new ideas for the treatment of metabolic diseases.

Studies have shown that microRNA can directly or indirectly regulate insulin signaling pathways (Trajkovski et al. 2011; Liu et al. 2014). The identification of abnormally expressed microRNAs that target important proteins in the insulin signaling pathway in the liver of diabetes patients, as well as the further exploration for their mechanisms in regulating insulin signaling pathway can increase our understanding regarding of the molecular mechanisms of hepatic insulin resistance and provide new ideas for treating type II diabetes. In this report, we constructed a model of insulin resistance by treating HepG2 cells with PA. At the same time, we also found PA can cause a decrease in the protein level of IR. This observation was consistent with previous studies (Yang et al. 2014). Using bioinformatics predictions we found there was a miR-15b binding site on the 3′ UTR of IR and it was conserved across species. We then used dual luciferase reporter assays to demonstrate that IR was a direct target gene for miR-15b. At the same time, we demonstrated it can affect the insulin sensitivity of cells by regulating the expression level of miR-15b in HepG2 cells. To further explore the effects of miR-15b on insulin sensitivity in vivo, we used AntagomiR-15b to reduce the expression of miR-15b in the liver of ob/ob mice. We found the protein level of IR increased significantly in the liver, and the sensitivity of mice to insulin was also significantly enhanced. These results indicated that aberrantly overexpressed miR-15b indeed suppresses the insulin signaling pathway by targeting IR.

Palmitate is a ligand for PPARα (Forman et al. 1997; Kliewer et al. 1997), so we hypothesis PA may promote miR-15b expression by activating PPARα. We cloned the promoter of miR-15b and constructed a luciferase reporter gene. We found the luciferase activity could be enhanced or suppressed by overexpressing or inhibiting PPARα respectively. Furthermore, inhibition of PPARα by transfected with shRNA in PA-treated HepG2 cell can aborted the enhancement of miR-15b level by PA. These results demonstrated PA could regulate the expression of miR-15b by activating PPARα and PPARα may also play a role in the development of insulin resistance. PPARα is a ligand-activated transcription factor that is abundantly expressed in liver (Escher et al. 2001). PPARα governs the hepatic expression of genes involved in nearly all aspects of lipid metabolism (Kersten 2014). Therefore, there are many studies on the correlation between PPARα and Non-alcoholic fatty liver disease (NAFLD) (Hashimoto et al. 2000; Francque et al. 2015), however, there are few studies on the relationship between PPARα and type II diabetes. Cariou B et al demonstrated GFT505, a dual peroxisome proliferator-activated receptor (PPAR)-α/δ agonist, can improve insulin sensitivity in mice (Cariou et al. 2013). At the same time, the expression level of PPARα in fatty liver was decreased (Francque et al. 2015), and fatty liver was also closely related to liver insulin resistance (Marchesini et al. 1999; Bugianesi et al. 2005; Targher et al. 2007). Therefore, we suspect there may be other mechanisms for regulating miR-15b.

In summary, our study demonstrated the functional importance of hepatic miR-15b in the regulation of insulin signaling pathway. Based on our findings, the PA-induced miR-15b regulated the liver insulin signaling pathway by targeting IR. Moreover, we identified a specific molecular mechanism that link miR-15b to hepatic insulin resistance and provided a potential new target for the treatment of type II diabetes.

## References

[CIT0001] Badman MK, Pissios P, Kennedy AR, Koukos G, Flier JS, Maratos-Flier E. 2007 Hepatic fibroblast growth factor 21 is regulated by PPARα and is a key mediator of hepatic lipid metabolism in Ketotic States. Cell Metab. 5:426–437. doi: 10.1016/j.cmet.2007.05.002 17550778

[CIT0002] Bartel DP. 2009 MicroRNAs: target recognition and regulatory functions. Cell. 136:215–233. doi: 10.1016/j.cell.2009.01.002 19167326 PMC3794896

[CIT0003] Boren J, Taskinen MR, Olofsson SO, Levin M. 2013 Ectopic lipid storage and insulin resistance: a harmful relationship. J Intern Med. 274:25–40. doi: 10.1111/joim.12071 23551521

[CIT0004] Bugianesi E, McCullough AJ, Marchesini G. 2005 Insulin resistance: a metabolic pathway to chronic liver disease. Hepatology. 42:987–1000. doi: 10.1002/hep.20920 16250043

[CIT0005] Cariou B, Hanf R, Lambert-Porcheron S, Zair Y, Sauvinet V, Noel B, Flet L, Vidal H, Staels B, Laville M. 2013 Dual peroxisome proliferator-activated receptor alpha/delta agonist GFT505 improves hepatic and peripheral insulin sensitivity in abdominally obese subjects. Diabetes Care. 36:2923–2930. doi: 10.2337/dc12-2012 23715754 PMC3781493

[CIT0006] Cimmino A, Calin GA, Fabbri M, Iorio MV, Ferracin M, Shimizu M, Wojcik SE, Aqeilan RI, Zupo S, Dono M, et al. 2005 miR-15 and miR-16 induce apoptosis by targeting BCL2. Proc Natl Acad Sci USA. 102:13944–13949. doi: 10.1073/pnas.0506654102 16166262 PMC1236577

[CIT0007] Escher P, Braissant O, Basu-Modak S, Michalik L, Wahli W, Desvergne B. 2001 Rat PPARs: quantitative analysis in adult rat tissues and regulation in fasting and refeeding. Endocrinology. 142:4195–4202. doi: 10.1210/endo.142.10.8458 11564675

[CIT0008] Forman BM, Chen J, Evans RM. 1997 Hypolipidemic drugs, polyunsaturated fatty acids, and eicosanoids are ligands for peroxisome proliferator-activated receptors alpha and delta. Proc Natl Acad Sci USA. 94:4312–4317. doi: 10.1073/pnas.94.9.4312 9113986 PMC20719

[CIT0009] Francque S, Verrijken A, Caron S, Prawitt J, Paumelle R, Derudas B, Lefebvre P, Taskinen MR, Van Hul W, Mertens I, et al. 2015 PPARα gene expression correlates with severity and histological treatment response in patients with non-alcoholic steatohepatitis. J Hepatol. 63:164–173. doi: 10.1016/j.jhep.2015.02.019 25703085

[CIT0010] Hashimoto T, Cook WS, Qi C, Yeldandi AV, Reddy JK, Rao MS. 2000 Defect in peroxisome proliferator-activated receptor α-inducible fatty acid oxidation determines the severity of hepatic steatosis in response to Fasting. J Biol Chem. 275:28918–28928. doi: 10.1074/jbc.M910350199 10844002

[CIT0011] Joshi RL, Lamothe B, Cordonnier N, Mesbah K, Monthioux E, Jami J, Bucchini D. 1996 Targeted disruption of the insulin receptor gene in the mouse results in neonatal lethality. EMBO J. 15:1542–1547. doi: 10.1002/j.1460-2075.1996.tb00498.x 8612577 PMC450062

[CIT0012] Kahn SE, Hull RL, Utzschneider KM. 2006 Mechanisms linking obesity to insulin resistance and type 2 diabetes. Nature. 444:840–846. doi: 10.1038/nature05482 17167471

[CIT0013] Kersten S. 2014 Integrated physiology and systems biology of PPARα. Mol Metab. 3:354–371. doi: 10.1016/j.molmet.2014.02.002 24944896 PMC4060217

[CIT0014] Kliewer SA, Sundseth SS, Jones SA, Brown PJ, Wisely GB, Koble CS, Devchand P, Wahli W, Willson TM, Lenhard JM, et al. 1997 Fatty acids and eicosanoids regulate gene expression through direct interactions with peroxisome proliferator-activated receptors alpha and gamma. Proc Natl Acad Sci USA. 94:4318–4323. doi: 10.1073/pnas.94.9.4318 9113987 PMC20720

[CIT0015] Liang T, Liu C, Ye Z, Xue Y. 2013 Deep sequencing of small RNA repertoires in mice reveals metabolic disorders-associated hepatic miRNAs. PLoS One. 8:e80774. doi: 10.1371/journal.pone.0080774 24260478 PMC3829963

[CIT0016] Liu W, Cao H, Ye C, Chang C, Lu M, Jing Y, Zhang D, Yao X, Duan Z, Xia H, et al. 2014 Hepatic miR-378 targets p110α and controls glucose and lipid homeostasis by modulating hepatic insulin signalling. Nat Commun. 5:5684. doi: 10.1038/ncomms6684 25471065

[CIT0017] Marchesini G, Brizi M, Morselli-Labate AM, Bianchi G, Bugianesi E, McCullough AJ, Forlani G, Melchionda N. 1999 Association of nonalcoholic fatty liver disease with insulin resistance. Am J Med. 107:450–455. doi: 10.1016/S0002-9343(99)00271-5 10569299

[CIT0018] Michael MD, Kulkarni RN, Postic C, Previs SF, Shulman GI, Magnuson MA, Kahn CR. 2000 Loss of insulin signaling in hepatocytes leads to severe insulin resistance and progressive hepatic dysfunction. Mol Cell. 6:87–97. doi: 10.1016/S1097-2765(05)00015-8 10949030

[CIT0019] Pandey AK, Verma G, Vig S, Srivastava S, Srivastava AK, Datta M. 2011 miR-29a levels are elevated in the db/db mice liver and its overexpression leads to attenuation of insulin action on PEPCK gene expression in HepG2 cells. Mol Cell Endocrinol. 332:125–133. doi: 10.1016/j.mce.2010.10.004 20943204

[CIT0020] Petersen KF, Shulman GI. 2006 Etiology of insulin resistance. Am J Med. 119(5):S10–S16. English. doi: 10.1016/j.amjmed.2006.01.009 16563942 PMC2995525

[CIT0021] Rottiers V, Näär AM. 2012 MicroRNAs in metabolism and metabolic disorders. Nat Rev Mol Cell Biol. 13:239–250. doi: 10.1038/nrm3313 22436747 PMC4021399

[CIT0022] Samuel VT, Shulman GI. 2012 Mechanisms for insulin resistance: common threads and missing links. Cell. 148:852–871. doi: 10.1016/j.cell.2012.02.017 22385956 PMC3294420

[CIT0023] Sun LL, Jiang BG, Li WT, Zou JJ, Shi YQ, Liu ZM. 2011 MicroRNA-15a positively regulates insulin synthesis by inhibiting uncoupling protein-2 expression. Diabetes Res Clin Pr. 91:94–100. doi: 10.1016/j.diabres.2010.11.006 21146880

[CIT0024] Targher G, Bertolini L, Padovani R, Rodella S, Tessari R, Zenari L, Day C, Arcaro G. 2007 Prevalence of nonalcoholic fatty liver disease and its association with cardiovascular disease among type 2 diabetic patients. Diabetes Care. 30:1212–1218. doi: 10.2337/dc06-2247 17277038

[CIT0025] Trajkovski M, Hausser J, Soutschek J, Bhat B, Akin A, Zavolan M, Heim MH, Stoffel M. 2011 MicroRNAs 103 and 107 regulate insulin sensitivity. Nature. 474:649–653. doi: 10.1038/nature10112 21654750

[CIT0026] Xia L, Zhang DX, Du R, Pan YL, Zhao LN, Sun SR, Hong L, Liu J, Fan DM. 2008 miR-15b and miR-16 modulate multidrug resistance by targeting BCL2 in human gastric cancer cells. Int J Cancer. 123:372–379. doi: 10.1002/ijc.23501 18449891

[CIT0027] Yang WM, Jeong HJ, Park SY, Lee W. 2014 Saturated fatty acid-induced miR-195 impairs insulin signaling and glycogen metabolism in HepG2 cells. FEBS Lett. 588:3939–3946. doi: 10.1016/j.febslet.2014.09.006 25240198

[CIT0028] Yue JM, Tigyi G. 2010 Conservation of miR-15a/16-1 and miR-15b/16-2 clusters. Mamm Genome. 21:88–94. doi: 10.1007/s00335-009-9240-3 20013340 PMC2820079

